# Association of TRAIL and Its Receptors with Large-Artery Atherosclerotic Stroke

**DOI:** 10.1371/journal.pone.0136414

**Published:** 2015-09-03

**Authors:** Xudong Pan, Meng Pang, Aijun Ma, Kun Wang, Zhang Zhang, Qianwei Zhong, Shuna Yang

**Affiliations:** Department of Neurology, the Affiliated Hospital of the Qingdao University, Qingdao, Shandong Province 266100, PR China; IIBB-CSIC-IDIBAPS, SPAIN

## Abstract

**Objective:**

To investigate the association of the tumor necrosis factor-related apoptosis-inducing ligand (TRAIL) and its receptors, osteoprotegerin (OPG) and death receptor 5 (DR5) with large-artery atherosclerosis (LAA) stroke and its prognosis.

**Methods:**

We included patients with LAA stroke (n = 132) according to the TOAST classification system and controls (n = 60). To evaluate the extent and severity of cerebral atherosclerosis, the LAA stroke group was subdivided into 3 subgroups by number of cerebral arteries with atherosclerotic stenosis (≥50%): single, double and multiple (≥3). Plasma levels of TRAIL, OPG and DR5 were measured by ELISA. Ordinal logistic regression was used to analyze the association between the plasma levels of TRAIL, OPG, DR5 and the severity of cerebral atherosclerosis. Prognosis was determined by the Modified Rankin Scale at 3 months after stroke. Receiver operating characteristic (ROC) curve was used to evaluated TRAIL as a predictor of prognosis.

**Results:**

Plasma TRAIL level was significantly lower for LAA patients than controls (*P*<0.001), while plasma OPG and DR5 levels were higher (both *P*<0.001). Logistic regression analysis revealed that risk of severe cerebral atherosclerosis was reduced significantly with increased plasma level of TRAIL (*OR* 0.438; 95% *CI* 0.282–0.681; *P*<0.001), whereas increased with high plasma levels of OPG and DR5 (*OR* 2.707; 95% *CI* 1.702–4.302, *P* <0.001; *OR* 3.593; 95% *CI* 1.878–6.869, *P* <0.001). Plasma TRAIL level was negatively correlated with the prognosis (r = - 0.372, *P* <0.001). The optimal cut-off value of TRAIL for prognosis was 848.63 pg/mL. The sensitivity and specificity at this cut-off value were 63.1% and 86.2%, respectively. After adding the plasma TRAIL level into the multivariate model of ROC, the area under the ROC curve was increased from 0.639 to 0.785, but the change was not statistical significant (*P* = 0.146).

**Conclusions:**

TRAIL and its receptors OPG and DR5 may be involved in LAA stroke and the plasma level of TRAIL may be a biomarker predicting the severity of cerebral atherosclerosis and the prognosis of LAA stroke.

## Introduction

Tumor necrosis factor (TNF)-related apoptosis-inducing ligand (TRAIL) belongs to the TNF ligand super family, which was first discovered and successfully cloned by Wiley et al. [1] in 1995. As a death receptor for TRAIL, death receptor 5 (DR5), cloned by Pan et al. [2] in 1997, triggers TRAIL-induced apoptosis after binding to TRAIL. Osteoprotegerin (OPG), as a decoy receptor for TRAIL, blocks TRAIL-induced apoptosis by inhibiting TRAIL binding to the death receptor [3].

TRAIL and its receptors are expressed in various cells such as endothelial cells (ECs), vascular smooth muscle cells (VSMCs) and some inflammatory cells (lymphocytes, neutrophils, mast cells), and are directly or indirectly involved in the pathogenesis of atherosclerosis [4–6]. Some studies have explored the roles of plasma levels of TRAIL and its receptors in atherosclerosis [7–9]. Large-artery atherosclerosis (LAA) stroke is a common type of cerebrovascular atherosclerosis according to the Trial of Org 10172 in Acute Stroke Treatment (TOAST) classification [10]. Nevertheless, studies on TRAIL and its receptors (DR5, OPG) in cerebrovascular atherosclerosis are relatively few. The aims of present study were mainly to investigate the association of plasma levels of TRAIL and its receptors with LAA stroke, and explore the relationship between plasma TRAIL and prognosis of LAA stroke as well.

## Materials and Methods

### Subject selection and biological samples

We enrolled patients with LAA stroke according to the TOAST classification [10] admitted to the Department of Neurology at the Affiliated Hospital of Qingdao University within 7 days after stroke onset. All patients underwent CT and/or MRI of the brain, transcranial Doppler (TCD), carotid duplex ultrasonography, and CT or MR angiography of brain arteries. Digital subtraction angiography (DSA) was performed for patients with unconfirmed images. The extent and severity of cerebral atherosclerosis was determined by the number of major cerebral arteries with atherosclerotic stenosis (≥50%) or occlusion, which was used to divide LAA patients into 3 subgroups, with single, double and multiple (≥3) arteries involved.

The control subjects were selected from the Healthcare Clinic at the hospital during the same time. They were confirmed free from the previous history of stroke and infarcts through examination of brain CT or MRI, and without severe atherosclerosis or angiostenosis on TCD or cerebrovascular CT or MR angiography.

Subjects with other subtypes of stroke, severe heart disease and recent myocardial infarction or angina pectoris disorders, severe infections, severe nephrosis or liver disease, thrombotic diseases, or tumor were excluded.

The study was approved by the ethical committee of the Affiliated Hospital of Qingdao University (QDDXYXYFSYY-2014-005). All participants gave their informed consent to participate in this study.

Venous blood samples were drawn from the antecubital vein in all subjects after an overnight fast. For LAA patients, blood samples were obtained within 24 h of admission and isolated by centrifugation at 3000×g for 10 min; serum or plasma samples were aliquoted and stored at -70°C. All samples were thawed only once.

### TRAIL, OPG and DR5 measurements

Plasma levels of TRAIL, OPG and DR5 were measured with use of commercially ELISA kits (TRAIL OPG DR5 DuoSet; R&D Systems, Abingdon, UK). The detection ranges of the assays were 30–1000 pg/ml, 50–1500 ng/L, 20–1200pg/ml, respectively.

### Biochemical measurements

Levels of total cholesterol (TC), low-density lipoprotein (LDL), high-density lipoprotein (HDL), and triglycerides (TG), and glucose (GLU) were assessed by routine methods with a fully automatic biochemical analyzer (Hitachi 7600–020, Hitachi, Tokyo, Japan). Serum level of high sensitivity C-reactive protein (hs-CRP) was measured by immunoturbidimetric assay with the automatic biochemical analyzer.

### Follow-up

All patients continued taking oral medication regularly after discharged and were followed up at 3 months to assess recovery (including modified Rankin score [mRS]), risk of recurrent stroke, and adverse drug reactions, among other items.

### Statistical analysis

Statistical analysis was performed using SPSS 17.0 for Windows (SPSS Inc., Chicago, IL). Data for continuous variables were presented as means ± standard deviation (SD) and for categorical variables as frequency (percentage). Continuous variables were compared by the *t*-test or one-factor analysis of variance, and categorical variables by chi-square test. Data were analyzed by binary logistic regression with adjustment for hypertension, diabetes, smoking, and drinking alcohol. The association between the severity of cerebral atherosclerosis and plasma levels of TRAIL, OPG, and DR5 was evaluated by ordinal logistic regression. The correlation between TRAIL, OPG, DR5 levels and prognosis was determined by Spearman's correlation analysis. Receiver operating characteristic (ROC) curve analysis was used to examine predictors of TRAIL for prognosis. The optimal cut-off level of TRAIL was ascertained by the Youden index. To assess the added predictive ability of TRAIL for prognosis, we compared the area under the ROC curve (AUC) in the multivariate model with and without TRAIL. A two-tailed P value <0.05 was considered statistically significant.

## Results

### Patient data

We enrolled 132 patients with LAA (80 men; mean age 63.7±12.1 years) and 60 controls (31 men; mean age 60.2±12.1 years). Patients and controls did not differ in mean age, gender, presence of diabetes or coronary disease or TG and LDL levels (*P* >0.05). Frequency of hypertension, smoking, alcohol consumption was higher for patients than controls (*P* <0.05), and levels of TC, hs-CRP and GLU were higher (*P* <0.05). HDL level was lower for patients than controls (*P* <0.05, Table 1).

**Table 1:** Basic data for LAA stroke patients and the controls.

Variables	LAA patients (n = 132)	Controls (n = 60)	*P* value *
Age, year, mean±SD	63.7±12.1	60.2±12.1	0.062
Gender, male (%)	80(60.6)	31(51.7)	0.245
Hypertension, n (%)	102(77.3)	25(41.7)	0.000
Diabetes, n (%)	39(29.5)	10(16.7)	0.580
Coronary Disease, n (%)	27(20.5)	12(20)	0.942
Smoking, n (%)	62(46.9)	14(23.3)	0.002
Alcohol consumption, n (%)	66(50)	16(26.7)	0.002
TG,mmol/L, mean±SD	1.95±1.59	1.58±0.73	0.088
TC, mmol/L, mean±SD	4.76±0.98	4.17±1.05	0.000
HDL, mmol/L, mean±SD	1.05±0.23	1.21±0.26	0.000
LDL, mmol/L, mean±SD	2.38±0.83	2.59±0.77	0.107
hs-CRP, mg/L, mean±SD	16.86±27.92	6.91±21.92	0.009
GLU, mmol/L, mean±SD	6.25±2.59	5.34±1.33	0.002

* Differences between LAA and controls determined using chi-square test or *t*-test.

TG: triglycerides, TC: total cholesterol, HDL: high-density lipoprotein, LDL: low-density lipoprotein, hs-CRP: high sensitivity C-reactive protein, GLU: fasting blood-glucose

### Plasma levels of TRAIL, OPG and DR5

**Fig 1:**
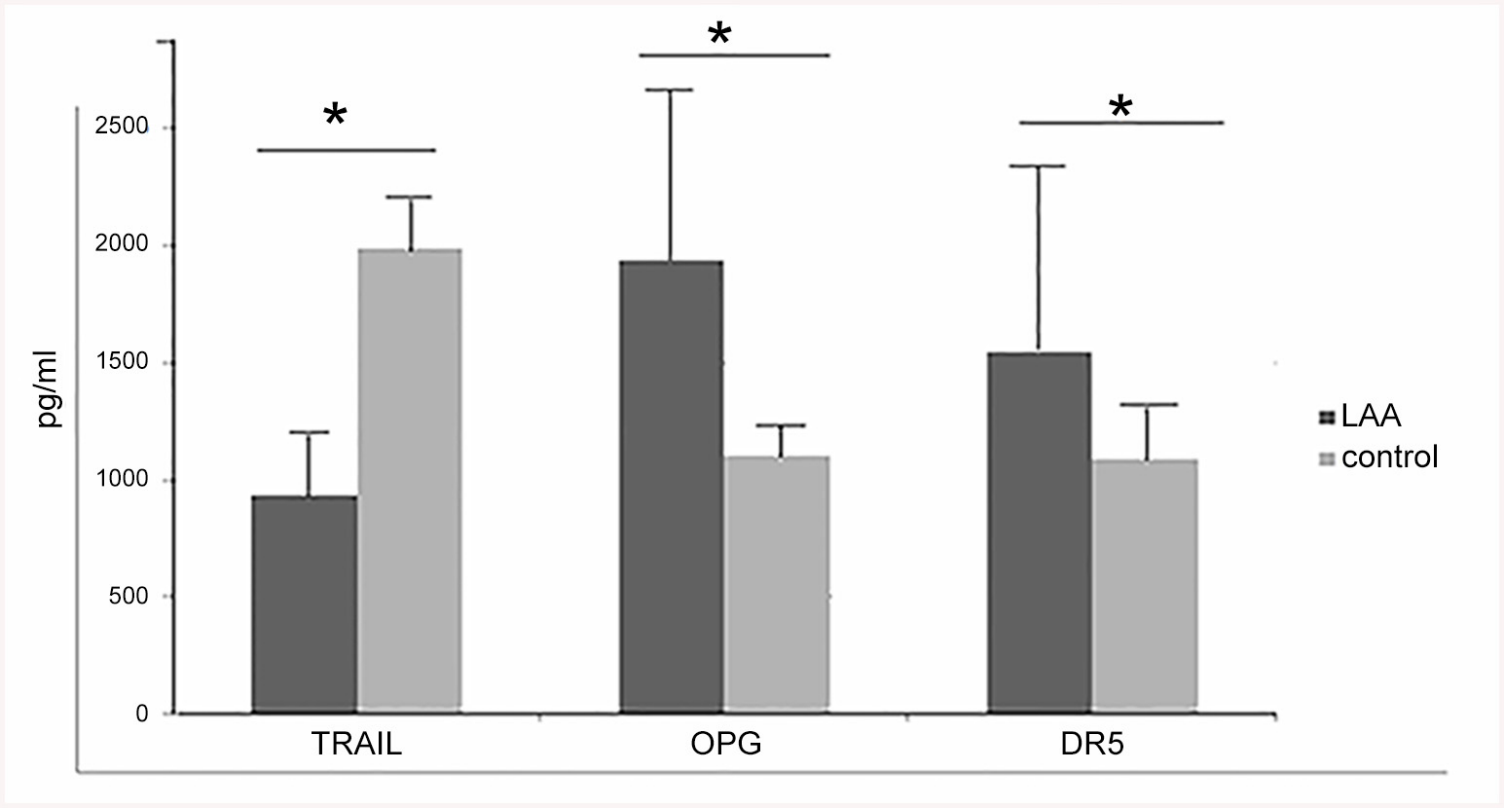
Comparison of the plasma levels of TRAIL, OPG and DR5 between LAA patients and controls. * *P* <0.001 by *t*-test.

With all these risk factors listed being adjusted by the binary logistic regression analysis, significant differences in the plasma levels of TRAIL, OPG and DR5 were noticed between LAA patients and controls (Fig 1). Plasma level of TRAIL was significantly lower for patients than controls (*t* = -23.129, *P* <0.001), whereas plasma levels of OPG and DR5 were higher (*t* = 11.186, *P* <0.001; *t* = 4.998, *P* <0.001, respectively).

### Correlation of plasma levels of TRAIL, OPG and DR5 with the extent and severity of cerebral atherosclerosis

Among 132 patients with LAA stroke, 42 had single-artery stenosis (≥50%), 38 double-artery stenosis and 52 multiple-artery stenosis (Table 2). Plasma levels of TRAIL, OPG and DR5 from these 3 subgroups were analyzed by ordinal logistic regression. Risk of severe cerebral atherosclerosis was reduced significantly with plasma level of TRAIL increased by one SD (*OR* 0.438; 95% *CI* 0.282–0.681; *P*<0.001), whereas increased with high plasma levels of OPG and DR5 (*OR* 2.707; 95% *CI* 1.702–4.302, *P* <0.001; *OR* 3.593; 95% *CI* 1.878–6.869, *P* <0.001; Tables 2 and 3).

**Table 2:** Plasma levels of TRAIL, DR5 and OPG in different subgroups of LAA.

Variables, pg/mL	Single-artery (n = 42)	Double-artery (n = 37)	Multiple-arterys (n = 53)	*P* value *
TRAIL	1067.27±408.61	873.85±335.71	861.20±138.03	0.003
OPG	1465.09±756.96	1863.98±663.95	2372.38±707.29	0.000
DR5	1053.32±323.93	1445.55±1116.94	2002.55±972.05	0.000

Data were presented as means ± SD.

* comparing the 3 subgroups by one-factor analysis of variance.

TRAIL, tumor necrosis factor (TNF)-related apoptosis-inducing ligand; OPG, osteoprotegerin; DR5, death receptor 5

**Table 3:** Logistic regression analysis of association of TRAIL, OPG, and DR5 levels and cerebral atherosclerosis in LAA patients.

TRAIL per SD (316 pg/mL)			OPG per SD (805pg/mL)			DR5 per SD (957pg/mL)		
*OR*	95%*CI*	*P* value	*OR*	95%*CI*	*P* value	*OR*	95%*CI*	*P* value
0.438	0.282–0.681	0.000	2.707	1.702–4.302	0.000	3.593	1.878–6.869	0.000

OR, odds ratio; 95% CI, 95% confidence interval

### Association of plasma level of TRAIL and the prognosis of LAA

Plasma level of TRAIL was negatively associated with mRS (r = -0.372, *P* <0.001), with no significant correlation between plasma levels of OPG, DR5 and the mRS (r = 0.053, *P* = 0.550; r = -0.025, *P* = 0.779).

**Fig 2:**
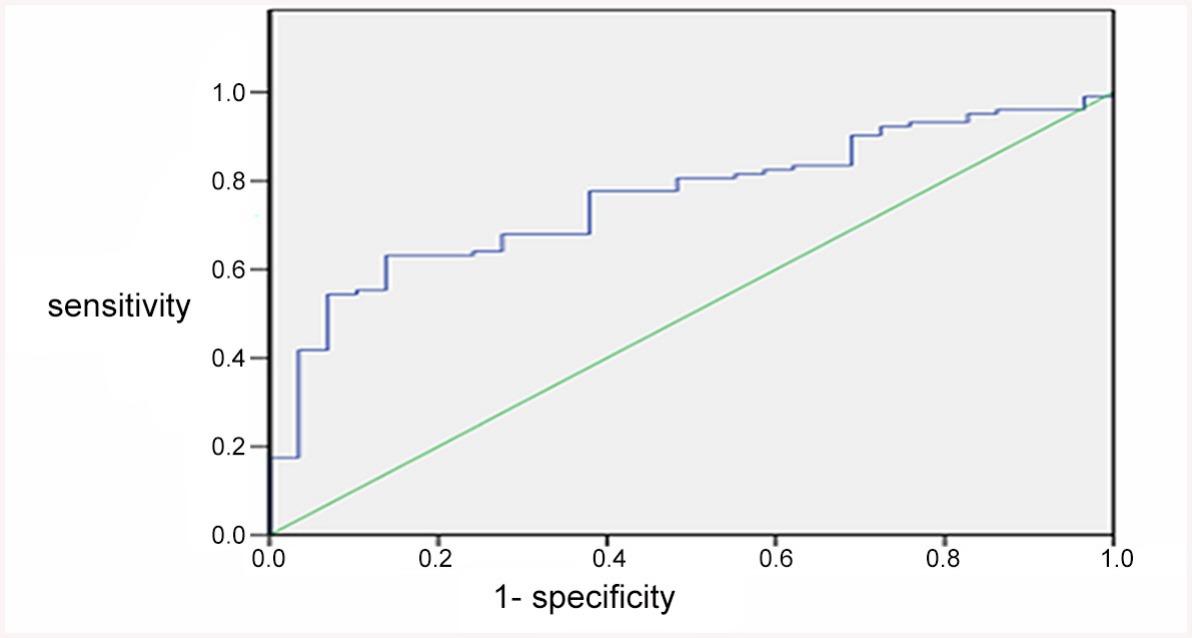
Receiver operating characteristic curve (ROC) analysis of TRAIL and the LAA prognosis. ROC of predicted sensitivity and 1-specificity with plasma levels of TRAIL alone. The area under the ROC curve (AUC) for TRAIL and mRS was 0.76, and the optimal cut-off value for TRAIL was 848.63 pg/mL.

**Fig 3:**
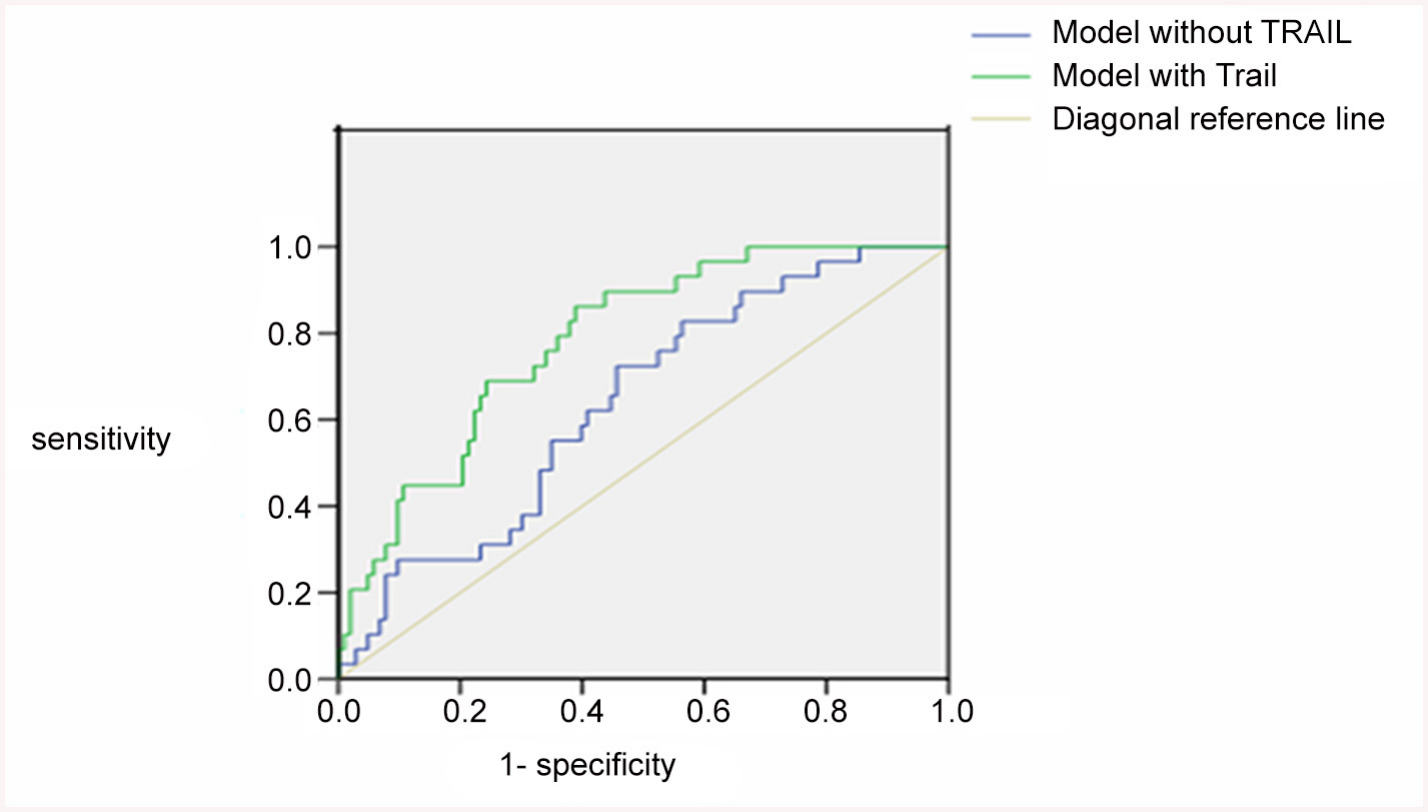
ROC analysis of the multivariate mode with TRAIL and LAA prognosis. ROC of predicted sensitivity and 1-specificity without TRAIL added to the multivariate model (AUC: 0.639). ROC with TRAIL added to the multivariate model (AUC: 0.785).

LAA patients were divided into those with good prognosis (mRS <3) and poor prognosis (mRS≥3). The AUC for the interaction of TRAIL level and mRS was 0.76 (Fig 2), and the optimal cut-off value for TRAIL was 848.63 pg/mL. The sensitivity of this cut-off value was 0.631 and the specificity 0.862. Adding the plasma TRAIL level into the multivariate model of variables for which LAA patients and controls differd, such as hypertension, smoking, alcohol consumption, and TC, hs-CRP, HDL and GLU levels, the AUC increased from 0.639 to 0.785 (Fig 3), but the change was not statistically significant (*P* = 0.146).

## Discussion

The present study showed that plasma level of TRAIL was significantly lower for patients with LAA than controls. Furthermore, the results from ordinal logistic regression suggested plasma level of TRAIL was significantly associated with the extent and severity of cerebral atherosclerosis. The plasma TRAIL levels decreased with more cerebral arteries involved in atherosclerotic stenosis. Similar results were also found in previous clinical studies: plasma levels of TRAIL were lower for patients with coronary artery disease and myocardial infraction than controls, and the plasma level of TRAIL decreased with increasing number of coronary arteries with atherosclerosis [7,10]. Thus, low TRAIL level may have a protective role in atherosclerosis.

It is well-known that inflammation and the immune response play significant roles in the occurrence and development of atherosclerosis, which is an important mechanism of LAA stroke [11]. ECs, VSMCs and some inflammatory cells such as lymphocytes and neutrophils are involved in the inflammatory response [12]. *In vitro* experiments showed that TRAIL induced apoptosis in ECs [13], VSMCs [14], lymphocytes [15] and neutrophils [16]. Nevertheless, that TRAIL could stimulate anti-apoptosis in ECs [17] and VSMCs [18] were also reported. Such contradictory effects in atherosclerosis may rely on the existence of five different types of receptors for TRAIL. Two death receptors, DR4/TRAIL-R1 and DR5/TRAIL-R2, initiate apoptosis; whereas the other three receptors, TRAIL-R3/DcR1, TRAIL-R4/DcR2 and OPG, act as decoy proteins for TRAIL binding [19]. However, an *in vivo* study in apoE-null mice showed that TRAIL could promote apoptosis in the plaque by binding to decoy receptors, thus slowing the progression of atherosclerosis [20].

In our study, OPG and DR5 levels were significantly higher in patients with LAA than controls and associated with the severity of cerebral atherosclerosis. Plasma OPG and DR5 levels increased with increasing number of arteries with atherosclerosis. With decreased plasma TRAIL level, OPG and DR5 may combine with it competitively. Correspondingly, increased TRAIL level strengthened the pro-apoptotic and anti-apoptotic effects. Eventually, the dynamic equilibrium was broken and the anti-apoptotic effects dominated. In a recent study, the plasma level of OPG was positively correlated with the severity of atherosclerosis in patients with cerebral infarction [21]. However, in this study plasma DR5 level was not studied.

With 3-month follow-up, plasma level of TRAIL was found negatively correlated with the prognosis of LAA as measured by mRS. However, plasma levels of OPG and DR5 were not associated with the prognosis. On AUC analysis, the optimal cut-off value for TRAIL was 848.63 pg/mL for assessing the prognosis of LAA stroke. *In vivo* study of apoE-null mice showed that increased plasma level of TRAIL could reduce the mortality [20]. TRAIL may promote plaque stabilization by preserving EC coverage, thereby decreasing the number of infiltrating macrophages and increasing that of VSMCs [20, 22]. Similarly, recent studies also demonstated that the OPG/TRAIL ratio and TRAIL level were associated with the poor outcome in patients with cardiovascular disease [23, 24].

In summary, in this case-control study, we found that TRAIL and its receptors, OPG and DR5 may be involved in the progression of LAA stroke. Plasma level of TRAIL might be of great value in predicting the prognosis of patients with LAA stroke. TRAIL level at 8.486pg/mL was the optimal cut-off value for prognosis. However, the number of cases in this study was limited, and patients were followed up for only 3 months. Further studies with a larger sample size and prolonged follow-up are required to estimate the utility of TRAIL level in the clinic.

## Supporting Information

S1 Text (XLS)
